# MAPK-activated protein kinase 2 is associated with poor prognosis of glioma patients and immune inhibition in glioma

**DOI:** 10.3389/fonc.2024.1307992

**Published:** 2024-01-23

**Authors:** Jinmin Sun, Sicheng Wu, Wenyu Zhao, Senrui Xue, Lei Zhang, Jing Ren

**Affiliations:** ^1^ Jiangsu Key Laboratory of Brain Disease Bioinformation, Research Center for Biochemistry and Molecular Biology, Xuzhou Medical University, Xuzhou, Jiangsu, China; ^2^ Laboratory of Clinical and Experimental Pathology, Department of Pathology, Xuzhou Medical University, Xuzhou, Jiangsu, China; ^3^ Department of Neurosurgery, The Affiliated Hospital of Xuzhou Medical University, Xuzhou, Jiangsu, China

**Keywords:** glioma, MAPKAPK2, prognosis, proliferation, migration

## Abstract

**Introduction:**

An effective therapeutic method to noticeably improve the prognosis of glioma patients has not been developed thus far. MAPK-activated protein kinase 2 (MAPKAPK2) is a serine/threonine kinase, which is involved in tumorigenesis, tumor growth, metastasis, and the inflammatory process. The clinical significance and molecular function of MAPKAPK2 in glioma remain unclear.

**Methods:**

MAPKAPK2 expression in human glioma tissues was detected by immunohistochemistry and analyzed from the transcriptome sequencing data in TCGA and CGGA. Prognostic nomogram was constructed to predict the survival risk of individual patients. GO and KEGG enrichment analyses were performed to analyze the function and pathways MAPKAPK2 involved. Single-cell RNA sequencing data was used to analyze the cell types in which MAPKAPK2 was enriched. Flow cytometry was used for cell cycle and apoptosis detection. The ability of cell proliferation and migration was analyzed by CCK8 and cell migration assay, respectively. Correlation analyses were performed to analyze the relationship of MAPKAPK2 with immune infiltration, immune regulators, chemokine, and chemokine receptors.

**Results:**

MAPKAPK2 was not only aberrantly upregulated in glioma tissues but also correlated with poor clinical characteristics. Moreover, MAPKAPK2 was prevalent in isocitrate dehydrogenase (IDH) wild-type and 1p/19q non-codeletion glioma cohorts and predicted poor prognosis of glioma patients. MAPKAPK2 may be involved in cell proliferation, cell migration, DNA damage repair, and immune regulation in glioma. MAPKAPK2 was enriched in microglia/macrophages and malignant tumor cells. Further investigation into cellular function revealed that inhibiting MAPKAPK2 suppressed the proliferation and migration of glioblastoma multiforme (GBM) cells in vitro. The inhibition of MAPKAPK2 significantly induced the G1 cell cycle arrest and cell apoptosis of GBM cells. Consistent with the enriched function of MAPKAPK2 in immune regulation, MAPKAPK2 was correlated with immune cell infiltration in glioma tissues. Mechanistically, a series of immune regulators, immunomodulatory chemokine, and chemokine receptors were positively correlated with MAPKAPK2 expression.

**Discussion:**

Our findings provide evidence of the clinical relevance of MAPKAPK2 in prognosis evaluation of glioma patients and highlight the underlying significance of MAPKAPK2 in glioma therapy.

## 1 Introduction

As the most lethal tumor, glioblastoma multiforme (GBM) which accounts for around 50%–60% of glioma still shows a dismal prognosis (1). Current traditional therapy methods for treating GBM are challenging. Molecular targeted therapy and immune therapy have been implicated in various other tumors, such as advanced melanoma and advanced non-small cell lung cancer, which brings a promising strategy for patients with GBM (2).

There are three structurally related mitogen-activated protein kinase (MAPK)-activated protein kinases (MAPKAPKs or MKs)—MAPKAPK2, MAPKAPK3, and MAPKAPK5 (3). MAPKAPK2, a prime downstream substrate of p38MAPK, is phosphorylated by p38 and functions in multiple pathways involving cell cycle regulation and cytokine and chemokine production, which control proliferation, angiogenesis, migration, and cell death (4, 5). Several studies revealed that MAPKAPK2 facilitated progression of diverse cancers by promoting the proliferation (6, 7), evasion (8), apoptosis induction (9), angiogenesis (10), or chemotherapy/radiotherapy resistance (11–13). MAPKAPK2 can regulate IL-1, IL-6, and chemokine expression in colitis-associated and spontaneous colon cancer models and promote the cancer progression. The chemokines induced by MAPKAPK2 signaling recruit macrophage influx to the colon cancer microenvironment and promotes colon cancer growth (14). In head and neck squamous cell carcinoma, inhibition of MAPKAPK2 reduced the production of inflammatory cytokines including IL-1α, IL-1β, and IL-6 induced by radiotherapy (6).

MAPKAPK2 also functions in tumor-associated macrophage polarization and promotes the macrophages into pro-tumorigenic M2-like macrophages to promote tumor progression in the colorectal cancer model (15). MAPKAPK2 also mediates the macrophage function by cytokine production in pancreatic neuroendocrine tumors. Macrophages with MAPKAPK2 depletion present increased cytotoxicity to pancreatic neuroendocrine tumors (16). In epidermal growth factor receptor (EGFR) vIII GBM cells, the p38-MAPKAPK2-human antigen R (HuR) pathway could enhance IL-6 secretion induced by IL-1β (17). MAPKAPK2 is also involved in chemotherapy (gemcitabine) resistance and radiation resistance in pancreatic cancer (11, 18) and head and neck squamous cell carcinoma (6). However, the clinical significance and detailed function of MAPKAPK2 in GBM remain unclear to date. GBM has a complex immunosuppressive environment due to the inter- or intratumor heterogeneity. Exploration of potent immune regulators is under eager need to improve the immune therapy of GBM patients.

In the present study, we show that MAPKAPK2 is aberrantly expressed in high-grade glioma tissues especially GBM. We assess the prognostic value of MAPKAPK2 in glioma and the biological function in GBM cells. Enriched function and signal pathways upon MAPKAPK2 expression indicate that MAPKAPK2 may participate in the progress of cell proliferation, cell migration or invasion, DNA damage repair, and immune regulation. Our data provide new insights into the role of MAPKAPK2 in the progression of glioma and present a promising strategy for glioma therapy involving MAPKAPK2 blockade.

## 2 Materials and methods

### 2.1 Clinical specimens and data acquisition

Glioma tissue samples (n = 93) and para-tumor tissues (n = 25) were obtained from the pathological department of the Affiliated Hospital of Xuzhou Medical University between, 2016 and, 2017. All samples were diagnosed by two pathologists.

The relevant RNA-seq data of gliomas were obtained from three databases: The Cancer Genome Atlas (TCGA) database (n = 703), the Genotype-Tissue Expression (GTEx, n = 1,157), and the Chinese Glioma Genome Atlas (CGGA) database (batch I, n = 413; batch II, n = 273). Cases with complete clinical information were carefully selected, including details such as age, gender, grade, histological type, survival time, chemotherapy or radiotherapy treatment, *IDH* status, 1p/19q codeletion status, and O6-methylguanine-DNA methyltransferase promoter methylation status in CGGA.

### 2.2 Immunohistochemistry analysis

The glioma tissues underwent a series of deparaffination and rehydration procedures. Subsequently, antigen retrieval was performed in citrate buffer using high pressure. After removing the endogenous peroxidase with 3% hydrogen peroxide for 10 minutes and blocking with 10% normal goat serum, the sections were incubated with the primary antibody (MAPKAPK2, 1:150, Proteintech, USA) overnight at 4°C. The primary antibody was subsequently discarded, and the sections were washed with PBS three times for 3 min each. The sections were incubated with horseradish peroxidase conjugated second antibody (PV6000, Zsgb Bio, China) at 37°C for 1 h, followed by washing with PBS three times for 3 min each. The staining was performed using diaminobenzidine for 3 min–5 min. Immunohistochemistry (IHC) staining was analyzed using the immunohistochemistry reactive score, which was the product of the positive staining intensity and area. The positive intensity was scored as 0 (negative), 1 (weak), 2 (moderate), or 3 (strong). The positive area was scored as 1 (0%–24%), 2 (25%–49%), 3 (50%–74%), or 4 (75%–100%). Based on the multiplied scores, the expression of MAPKAPK2 was classified into low expression group (0–4) and high expression group (6–12) for the analysis of MAPKAPK2 expression in patients with different clinical and pathological characteristics.

### 2.3 Cell culture and treatment

The GBM cells (U118, U251, U87, T98G, and LN229) and normal human astrocyte (NHA) were purchased from ATCC and cultured in high-glucose Dulbecco’s modification of Eagle’s medium (DMEM, Keygen Biotech, China) with 10% fetal bovine serum (FBS, Life Technologies, USA) at 37°C with 5% CO_2_. The selective MAPKAPK2 inhibitor (MK2-IN-1 hydrochloride) was purchased from MedChemExpress (HY-12834A).

### 2.4 Analyses of the relationships between MAPKAPK2 expression and prognosis and clinical phenotypes

Expression analyses of MAPKAPK2 in glioma and para-tumor tissue were performed from datasets downloaded from TCGA and CGGA. The MAPKAPK2 expression in pan-cancer and corresponding normal tissue was analyzed from the dataset of TCGA and GTEx. The Gene Expression Profiling Interactive Analysis web server (http://gepia.cancer-pku.cn/detail.php) was utilized to explore the gene expression profiles across all tumor samples and their corresponding normal tissues. Survival analysis was conducted using the Kaplan–Meier method and Cox regression with data obtained from TCGA and CGGA datasets. The R packages “survival” and “survminer” were utilized to analyze the data and generate survival curves. The receiver operating characteristic (ROC) analysis for the diagnostic value of MAPKAPK2 was conducted using R packages “pROC” and “ggplot2.

### 2.5 Immunoblot

GBM cells and NHA were harvested and lysed in RIPA buffer. Protein concentrations were analyzed by the BCA Protein Assay Kit from Beyotime (Shanghai, China). Polyacrylamide gel electrophoresis was performed to separate the total protein followed by transferring onto the nitrocellulose membranes from Millipore (Burlington, MA, United States). Subsequently, the membrane was put in 3% bovine serum albumin (BSA) for blocking at room temperature for 2 h. Primary antibodies against MAPKAPK2 (1:1,000 dilution; Santa Cruz, CA, United States) and GAPDH (1:10,000 dilution; Proteintech, Rosemont, IL, United States) were incubated with the membrane at 4°C overnight. Horseradish peroxidase-conjugated secondary antibodies were then used, and the proteins were detected using enhanced chemiluminescence reagent.

### 2.6 Univariate and multivariate Cox analyses

Univariate and multivariate Cox analyses were conducted using the R package “survival.” The analyses included MAPKAPK2 expression, WHO grade, 1p/19q codeletion, *IDH* status, primary therapy outcome, gender, and age.

### 2.7 Function and signal pathway enrichment analysis

We used Pearson’s correlation coefficients (|r| > 0.4, p < 0.001) to screen for genes that are coexpressed with MAPKAPK2 in TCGA database. The R package “clusterProfiler” was then used to perform enrichment analyses on MAPKAPK2 and the coexpressed genes, utilizing Gene Ontology (GO) and Kyoto Encyclopedia of Genes and Genomes (KEGG). In order to shed light on the disparity in survival, functions, and pathways between the groups with high and low expressions of MAPKAPK2, gene set enrichment analysis (GSEA) was performed using the R package “clusterProfiler.” We deemed values with |NES| > 1, p < 0.05, and false discovery rate (FDR) < 0.25 as statistically significant.

### 2.8 Relationships between MAPKAPK2 expression and immune infiltration

The levels of immune cell infiltration in glioma were measured using the “GSVA” R package, which combines single-sample GSEA (ssGSEA) algorithms. To obtain accurate estimates of immune infiltration, the “Immunedeconv” R package, which incorporates xCell algorithms, was employed. The Tumor and Immune System Interaction Database (TISIDB; http://cis.hku.hk/TISIDB) was utilized to examine the correlation between MAPKAPK2 expression and immune regulators, chemokines, and chemokine receptors in various human cancers.

### 2.9 Single-cell RNA sequencing analyses

Data from single-cell RNA-seq were gathered from the Broad Institute Single-Cell Portal in study 1 (https://singlecell.broadinstitute.org/single_cell/study/SCP393/single-cell-rna-seq-of-adult-and-pediatric-glioblastoma#study-download) and study 2 (https://singlecell.broadinstitute.org/single_cell/study/SCP50/single-cell-rna-seq-analysis-of-astrocytoma).

### 2.10 Flow cytometry

For cell-cycle detection, U87 and LN229 cells (1 × 10^6^) were treated with IN-1 (20 μM) for 48 h and then collected and stained with the cell cycle kit (KGA512, Keygen Biotech, China). The staining process involved fixing with icy ethyl alcohol overnight, washing, staining the cells with propidium iodide (PI), and analyzing by flow cytometry (FCM) (FACSCanto II, Germany) immediately. To analyze cell apoptosis, the U87 and LN229 cells (1 × 10^5^) were treated with the cell apoptosis kit (FMSAV647, Fcmacs, China) according to the provided instructions. Specifically, 5 μL Annexin V-Alexa Fluor 647 and 10 μL PI in 100 μL of 1× binding buffer were incubated with the collected cells. After incubation for 15 min in the dark, the samples were analyzed by flow cytometry (FACSCanto II, Germany) immediately.

### 2.11 Cell viability

U87 and LN229 cells (5,000 cells per well) were seeded in a 96-well plate with IN-1 (20 μM) in five replicates. After 24 h, 48 h, and 96 h, the viability of the cells was determined using the CCK8 kit (VC5001, VICMED) following the protocol. The absorbance value of A450 was measured using a microplate reader.

### 2.12 Cell migration assay

U87 and LN229 cells were treated with IN-1 for 48 h before starving for 16 h and then were seeded in up-chamber of the Transwell chamber in 100 μL DMEM free of serum (1 × 10^5^ per well). 500 μL DMEM including 10% fetal bovine serum was added in the lower chamber. The up-chambers were taken out after 24 h and were fixed with 4% paraformaldehyde. The up-chambers were stained with crystal violet for 20 min at room temperature. The photographs were taken by an Olympus microscope.

### 2.13 Statistical analysis

GraphPad Prism 7.0 (GraphPad Software, Inc., San Diego, CA, United States) and SPSS16.0 software (SPSS 16.0; SPSS, Inc., Chicago, IL, United States) were used for the statistical analyses. R (v.3.6.3) was employed to merge statistical data obtained from TCGA database. The chi-square (*χ* ^2^) test, Pearson’s correlation, or Spearman’s correlation analysis was performed for the correlation analysis. Survival data from the CGGA database were analyzed using the Kaplan–Meier method. The differences between two groups were analyzed by Student’s *t* test and Mann−Whitney *U* tests. One-way ANOVA and the Kruskal–Wallis test were conducted for analyzing the differences in more than two groups. *p* value < 0.05 was considered statistically significant (*p < 0.05, **p < 0.01, ***p < 0.001, ****p < 0.0001).

## 3 Results

### 3.1 MAPKAPK2 are aberrantly overexpressed in high-grade glioma, especially GBM

**Figure 1:**
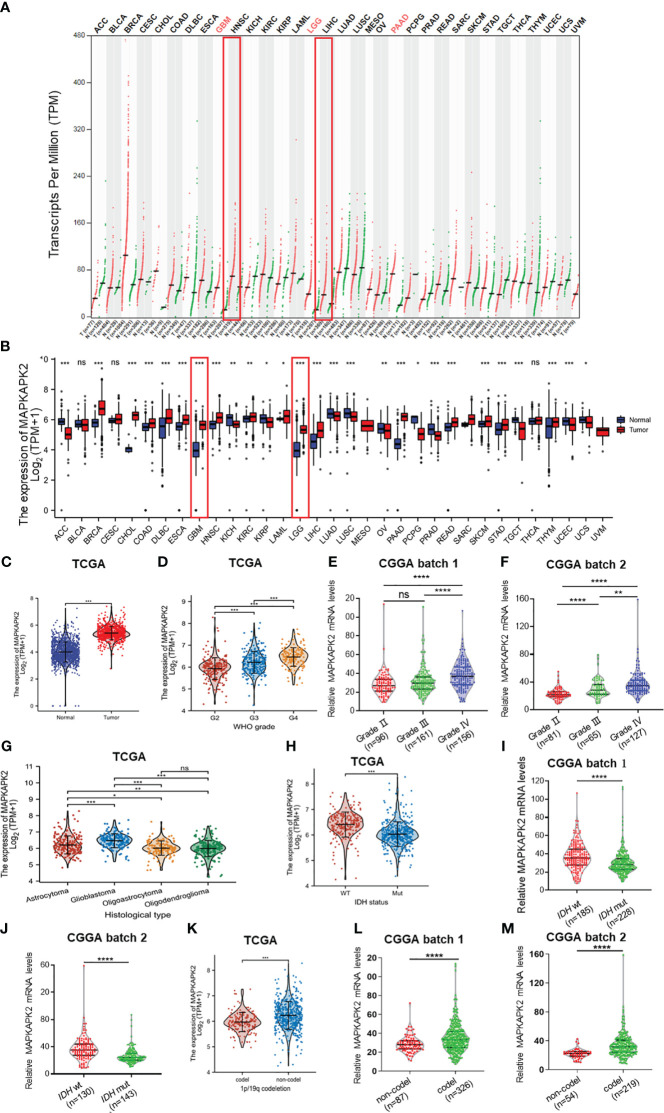
*MAPKAPK2* mRNA levels are aberrantly elevated in glioma tissue especially GBM. **(A)** Dot plot exhibiting the expression levels of *MAPKAPK2* in tumor samples and relevant normal tissues from the Gene Expression Profiling Interactive Analysis (GEPIA) web server. T, tumor tissue; N, normal tissue. **(B)** Comparison of *MAPKAPK2* expression among 33 types of tumors and corresponding normal samples from TCGA and Genotype-Tissue Expression (GTEx) databases. **(C)** The mRNA levels of *MAPKAPK2* in glioma and matched normal tissues from TCGA database. **(D–G)** The expression levels of *MAPKAPK2* in different WHO grades from TCGA **(D)**, CGGA batch 1 **(E)**, CCGA batch2 **(F)**, and different histological types **(G)**. **(H–M)** The expression levels of MAPKAPK2 in different isocitrate dehydrogenase (*IDH*) genotypes **(H–J)** and 1p/19q status **(K–M)** from TCGA and CGGA. *p<0.05, **p<0.01, ***p<0.001, **** p<0.0001, ns, no significance.

In order to unearth the potential roles of MAPKAPK2 in tumor progression, we firstly analyzed its expression in 33 types of human cancers and the corresponding normal tissues from TCGA. The results showed that *MAPKAPK2* was overexpressed in glioma including GBM and LGG compared with the normal brain tissues (**Figures 1A–C**). Further analyses of *MAPKAPK2* expression in glioma tissues from multiple datasets of TCGA and CGGA indicated that *MAPKAPK2* levels were higher in high-grade glioma especially GBM (**Figures 1D–F**). Correspondingly, *MAPKAPK2* expression analyses in different histological types also showed that *MAPKAPK2* levels were elevated in GBM compared with astrocytoma, oligoastrocytoma, and oligodendroglioma (**Figures 1G**). The expression of *MAPKAPK2* was further analyzed in different molecular subtypes including the *IDH* genotype and 1p/19q co-deletion status. We found that *MAPKAPK2* expression was predominantly enriched in *IDH* wild-type (wt) and 1p/19q non-codeletion glioma of which molecular subtypes predict poor prognosis of glioma patients from multiple datasets of TCGA and CGGA (**Figures 1H–M**).

**Figure 2:**
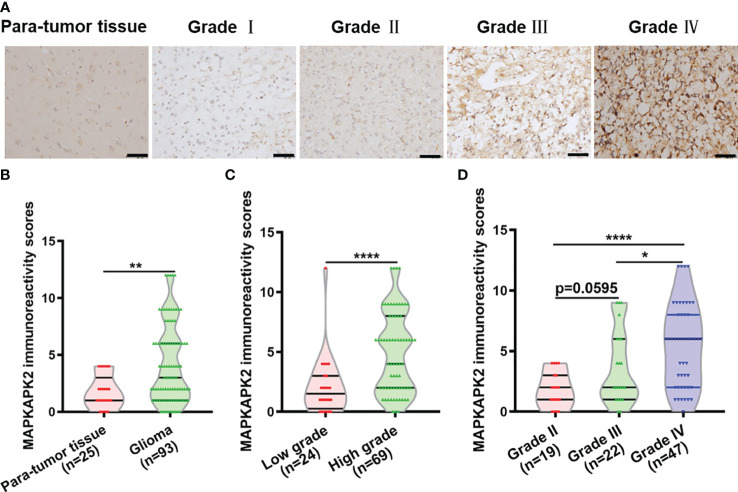
MAPKAPK2 protein is overexpressed in glioma tissues. **(A)** Representative immunohistochemistry (IHC) images of MAPKAPK2 in human glioma tissues with different grades and para-tumor tissues. Scale bar, 50 μm. **(B)** MAPKAPK2 immunoreactivity scores analyzed in human glioma tissues and para-tumor tissues. **(C)** MAPKAPK2 immunoreactivity scores analyzed in low-grade (grade I and grade II) and high-grade glioma tissues (grade III and grade IV). **(D)** MAPKAPK2 immunoreactivity scores analyzed in grade II, grade III, and grade IV glioma tissues. *p<0.05, **p<0.01, **** p<0.0001.

Further analyses of the relationship of *MAPKAPK2* mRNA with the clinical pathological characters of glioma patients showed that *MAPKAPK2* mRNA levels correlated with glioma grades, *IDH* genotype, 1p/19q co-deletion status, and the age of patients (**Table 1**). To further confirm the expression and relationship of MAPKAPK2 levels with these clinical pathological characters, we performed IHC detection of MAPKAPK2 in 93 glioma tissues and 25 para-tumor tissues (**Figure 2A**). The MAPKAPK2 protein levels were significantly elevated in glioma tissues compared with the para-tumor tissues and were obviously higher in GBM compared with low-grade glioma (**Figures 2B–D**, **Table 2**).

**Table 1:** Correlation of MAPKAPK2 mRNA with clinical characters of glioma patients in TCGA dataset.

Characteristic	Low expression of *MAPKAPK2*	High expression of *MAPKAPK2*	*р*
n	348	348	-
WHO grade, n (%)			<0.001
G2	164 (25.8%)	60 (9.4%)	
G3	115 (18.1%)	128 (20.2%)	
G4	31 (4.9%)	137 (21.6%)	
*IDH* status, n (%)			<0.001
WT	53 (7.7%)	193 (28.1%)	
Mut	290 (42.3%)	150 (21.9%)	
1p/19q codeletion, n (%)			<0.001
codel	122 (17.7%)	49 (7.1%)	
non-codel	225 (32.7%)	293 (42.5%)	
Age, median (IQR)	41 (33, 52)	52 (36, 62)	<0.001

WHO, World Health Organization; IDH, isocitrate dehydrogenase; WT, wild type; Mut, mutant; IQR, interquartile range.

**Table 2:** Relationship of MAPKAPK2 protein levels with the clinicopathological characteristics of 93 glioma patients detected by IHC.

Variable	Number (n)	MAPKAPK2 staining			
		Low (%)	High (%)	*Χ* ^2^	*p*
Gender				0.824	0.364
Male	56	37 (66.1)	19 (33.9)		
Female	37	21 (56.8)	16 (43.2)		
Age				0.001	0.981
<50 years	40	25 (62.5)	15 (37.5)		
≥50 years	53	33 (62.3)	20 (37.7)		
Tumor size				2.421	0.120
<5 cm	30	21 (70.0)	9 (30.0)		
≥5 cm	28	14 (50.0)	14 (50.0)		
WHO grade				15.437	0.000
Low (I–II)	24	23 (95.8)	1 (4.2)		
High (III–IV)	69	35 (50.7)	34 (49.3)		

WHO, World Health Organization.

These results indicate that MAPKAPK2 is positively correlated with the glioma malignant progression.

### 3.2 *MAPKAPK2* is correlated with poor prognosis of glioma patients

**Figure 3:**
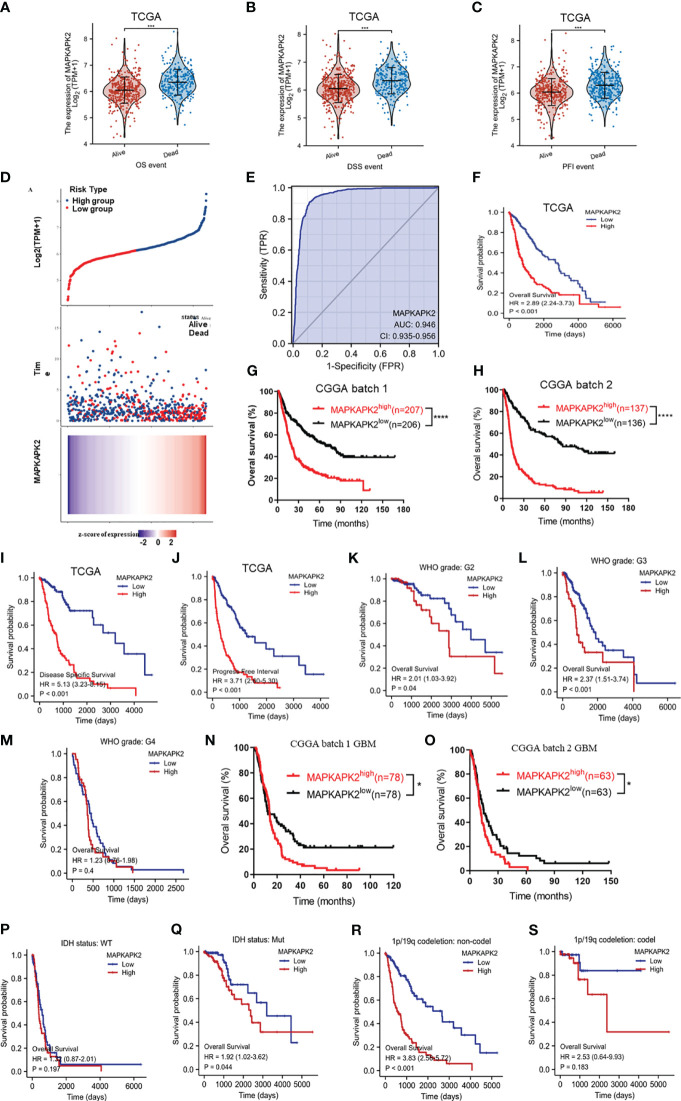
High *MAPKAPK2* levels are correlated with poor prognosis of glioma patients. **(A–C)** *MAPKAPK2* expression in glioma tissues of patients alive or dead from the analyses of overall survival (OS), disease-specific survival (DSS), and progression-free interval (PFI). **(D)** The risk score distribution, survival overview, and heatmap of glioma patients assorted into high- and low-risk groups upon *MAPKAPK2* expression from TCGA. **(E)** Receiver operating characteristic (ROC) curve of glioma tissues and normal brain tissues from TCGA to validate the diagnostic value of MAPKAPK2 in glioma patients. **(F-H)** The survival analyses of *MAPKAPK2* levels with OS of glioma patients in TCGA **(F)**, CGGA batch 1 **(G)**, and CGGA batch 2 **(H)**. **(I, J)** The survival analyses of *MAPKAPK2* levels with DSS **(I)** and PFI **(J)** of glioma patients from TCGA. **(K–S)** The survival analyses of *MAPKAPK2* levels with OS of glioma patients in different grades in TCGA **(K–M)**, CGGA batch 1 **(N)** and batch 2 **(O)**, different *IDH* genotypes **(P, Q)**, and 1p/19q codeletion status **(R, S)**. *p<0.05, ***p<0.001, **** p<0.0001.

In order to further explore the clinical significance of MAPKAPK2 in glioma, we investigated the prognostic value of *MAPKAPK2* in glioma by the informatic analyses from the datasets in TCGA and CGGA. *MAPKAPK2* levels were high in gliomas of dead patients rather than live patients according to overall survival (OS), disease-specific survival (DSS), and progression-free interval (PFI) (**Figures 3A–C**). Glioma patients in TCGA were divided into high- and low-risk groups according to the median value of *MAPKAPK2* expression as the cutoff point. More dead patients were present in the high-risk group compared with the low-risk group (**Figure 3D**). Analyses of the ROC curve showed that the area under the ROC curve (AUC) was 0.946, which indicated the high prognostic value of *MAPKAPK2* in glioma (**Figure 3E**). Overall survival analyses of glioma patients from TCGA and CGGA were performed according to the median value of *MAPKAPK2* as the cutoff point showed that high levels of *MAPKAPK2* were correlated with poor prognosis of glioma patients (**Figures 3F–H**). Consistently, high levels of *MAPKAPK2* also predicted poor prognosis of glioma patients in the DSS and PFI analyses from TCGA (**Figures 3I, J**).

**Figure 4:**
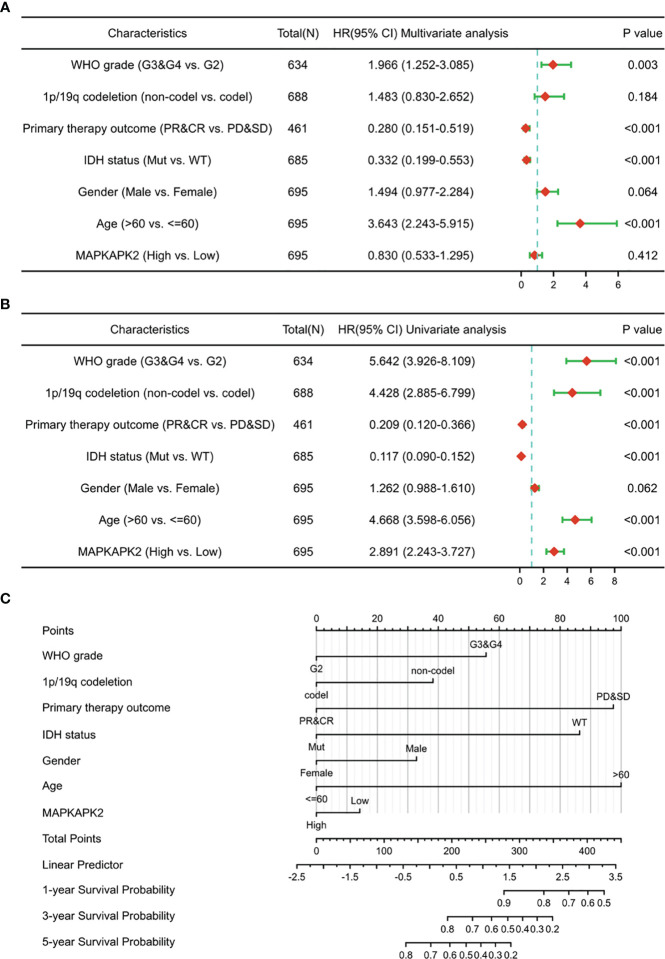
*MAPKAPK2* is a valuable prognosticator of survival for glioma patients. **(A, B)** Forest plot indicating univariate **(A)** and multivariate COX regression analyses **(B)** of *MAPKAPK2* with other clinicopathologic factors in glioma patients. **(C)** Nomogram for predicting 1‐, 3‐, and 5-year survival probability of glioma patients from TCGA.

We performed the overall survival analyses in different grades and different molecular subtypes of glioma to further explore the prognostic value of *MAPKAPK2*. High levels of *MAPKAPK2* correlated with poor prognosis of glioma patients with grade 2 and grade 3 glioma in TCGA and the patients with GBM in CGGA batches 1 and 2 (**Figures 3K, L–O**). Meanwhile, high levels of *MAPKAPK2* were positively correlated with poor prognosis of glioma patients with *IDH* mutant (mut) and 1p/19q non-codeletion gliomas but not in *IDH* wt and 1p/19q codeletion gliomas (**Figures 3P–S**). Next, we performed multivariate and univariate Cox risk proportional regression analyses to evaluate the prognosis risk of *MAPKAPK2*. Multivariate Cox regression analyses showed that WHO grades, 1p/19q codeletion status, and age were the risk factors of glioma, but there is no statistical significance of *MAPKAPK2* (**Figure 4A**). In univariate Cox regression analyses, *MAPKAPK2* was an independent prognostic factor in glioma (**Figure 4B**) in addition to WHO grades, 1p/19q codeletion status, and age. We also constructed a nomogram which included the *MAPKAPK2* expression and other independent prognostic factors to predict the 1-, 3-, and 5-year survival probability of glioma patients (**Figure 4C**).

These data indicate that MAPKAPK2 is positively correlated with poor prognosis of glioma patients and is a valuable prognostic indicator of glioma.

### 3.3 *MAPKAPK2*-related genes are mainly enriched in cell cycle, cell adhesion, DNA damage repair, and immune regulation

**Figure 5:**
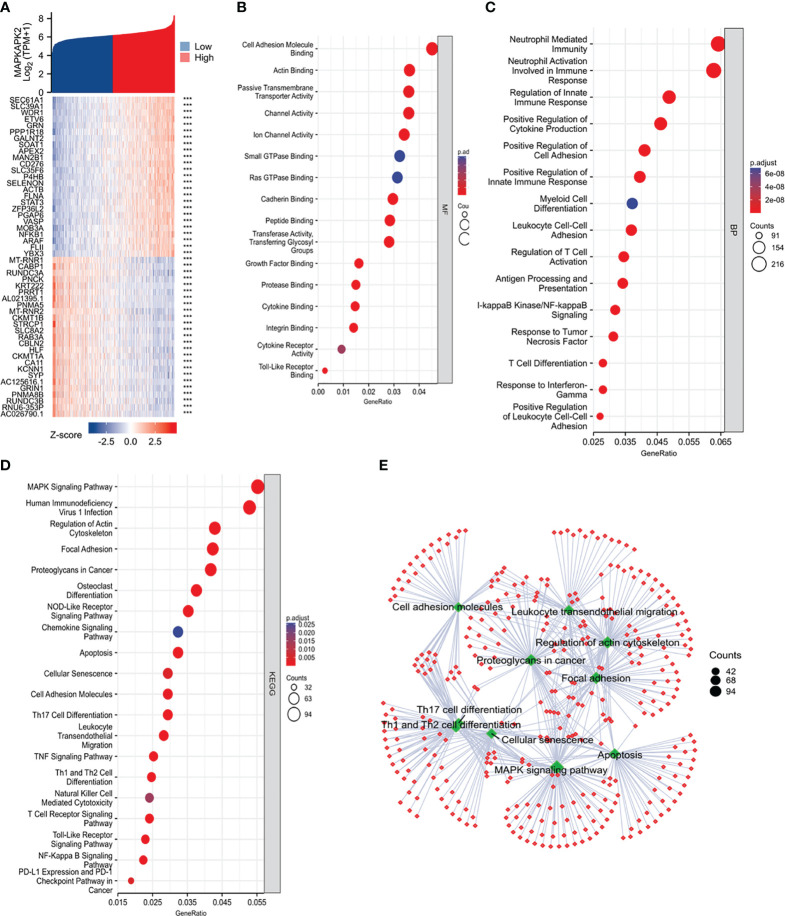
Function and pathway enrichment analyses of MAPKAPK2 in glioma from TCGA. **(A)** The heatmap of the top co-expressed 25 genes most positively or negatively correlated with *MAPKAPK2*. **(B, C)** GO enrichment analysis for molecular function (MF, **(B)**) and biological process (BP, **(C)**) of the related genes upon *MAPKAPK2* expression. **(D)** KEGG pathway enrichment analysis of the related genes upon *MAPKAPK2* expression. **(E)** The cnetplot of KEGG enrichment analysis of the related genes upon *MAPKAPK2* expression.

**Figure 6:**
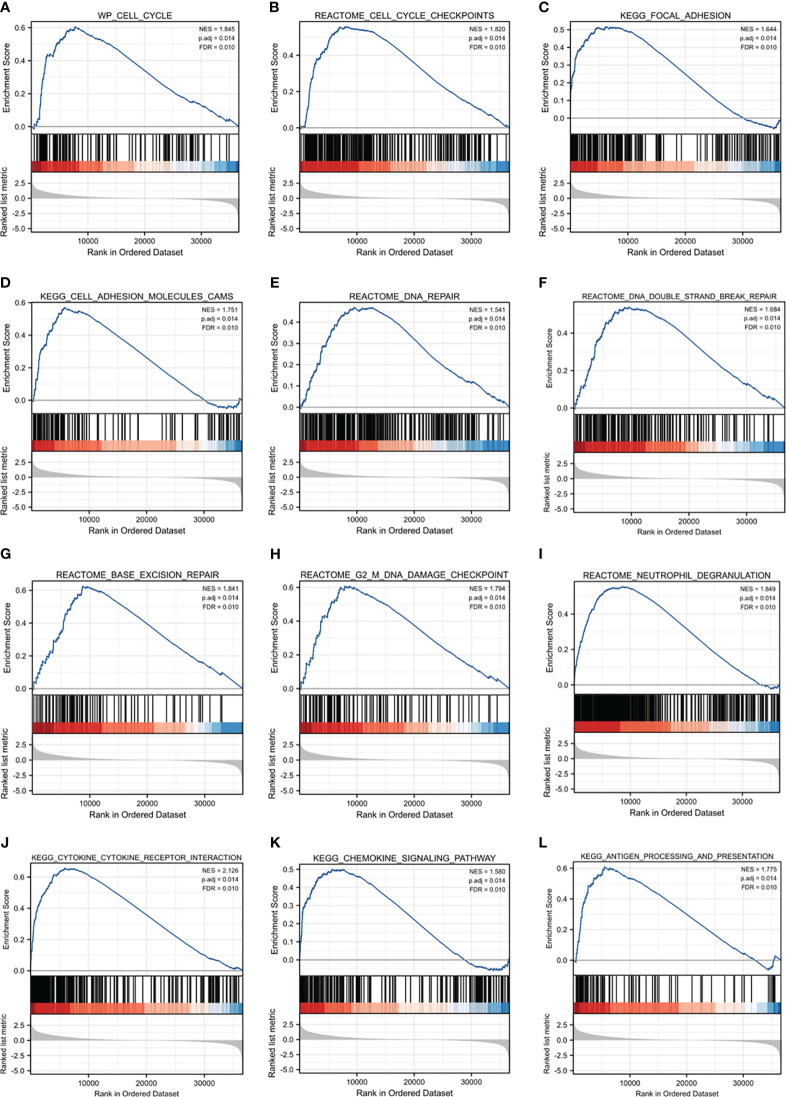
GSEA enrichment analyses of *MAPKAPK2* in glioma. **(A–L)** GSEA enrichment analyses indicated that cell cycle **(A)** and cell cycle checkpoint **(B)**, focal adhesion **(C)**, focal adhesion molecules **(D)**, DNA repair **(E)**, DNA double-strand break repair **(F)**, base excision repair **(G)**, G2/M DNA damage checkpoint **(H)**, neutrophil degranulation **(I)**, cytokine–cytokine receptor interaction **(J)**, chemokine signaling pathway **(K)**, and antigen progressing and presentation **(L)** were enriched.

To clarify the biological functions of MAPKAPK2, we firstly performed the correlation analysis between *MAPKAPK2* and the top 25 co-expressed genes positively or negatively related with *MAPKAPK2* (**Figure 5A**). Further enrichment analyses including GO enrichment analysis, KEGG signaling pathway enrichment analysis, and GSEA signaling enrichment pathway analysis were conducted to manifest the related function and signaling pathways of *MAPKAPK2* in glioma. Molecular function (MF) of GO enrichment analyses indicated that *MAPKAPK2* was associated with cell adhesion molecular binding, actin binding, etc. (**Figure 5B**). Biological progress (BP) of GO enrichment analyses showed that MAPKAPK2 was significantly correlated with neutrophil-mediated immunity, neutrophil activation involved in immune response, regulation of innate immune response, positive regulation of cytokine production, positive regulation of cell adhesion, myeloid cell differentiation, leukocyte cell–cell adhesion, regulation of T-cell activation, I-kappaB kinase/NF-kappaB signaling, T-cell differentiation, etc. (**Figure 5C**). KEGG signaling pathway enrichment analyses showed that the MAPK signal pathway was the top hit associated with *MAPKAPK2*. In addition, human immunodeficiency virus 1 infection, regulation of action cytoskeleton, focal adhesion, apoptosis, *PD-L1* expression, *PD-1* checkpoint pathway in cancer, etc., were enriched (**Figures 5D, E**). GSEA enrichment analyses indicated that multiple pathways were closely correlated with MAPKAPK2 expression in glioma. The enriched pathway included cell proliferation-associated signaling pathways such as cell cycle (**Figure 6A**) and cell cycle checkpoint (**Figure 6B**); cell adhesion signaling pathways such as focal adhesion (**Figure 6C**) and focal adhesion molecules (**Figure 6D**); DNA damage and repair-associated signaling pathways such as DNA repair (**Figure 6E**), DNA double-strand break repair (**Figure 6F**), base excision repair (**Figure 6G**), and G2/M DNA damage checkpoint (**Figure 6H**); and immune regulation-associated signaling pathways such as neutrophil degranulation (**Figure 6I**), cytokine–cytokine receptor interaction (**Figure 6J**), chemokine signaling pathway (**Figure 6K**), and antigen progressing and presentation (**Figure 6L**).

These analyses provide evidence that MAPKAPK2 may be enrolled in the proliferation, migration, DNA damage repair, and immune regulation of glioma.

### 3.4 MAPKAPK2 inhibition effectively suppresses the proliferation and migration of GBM cells

**Figure 7:**
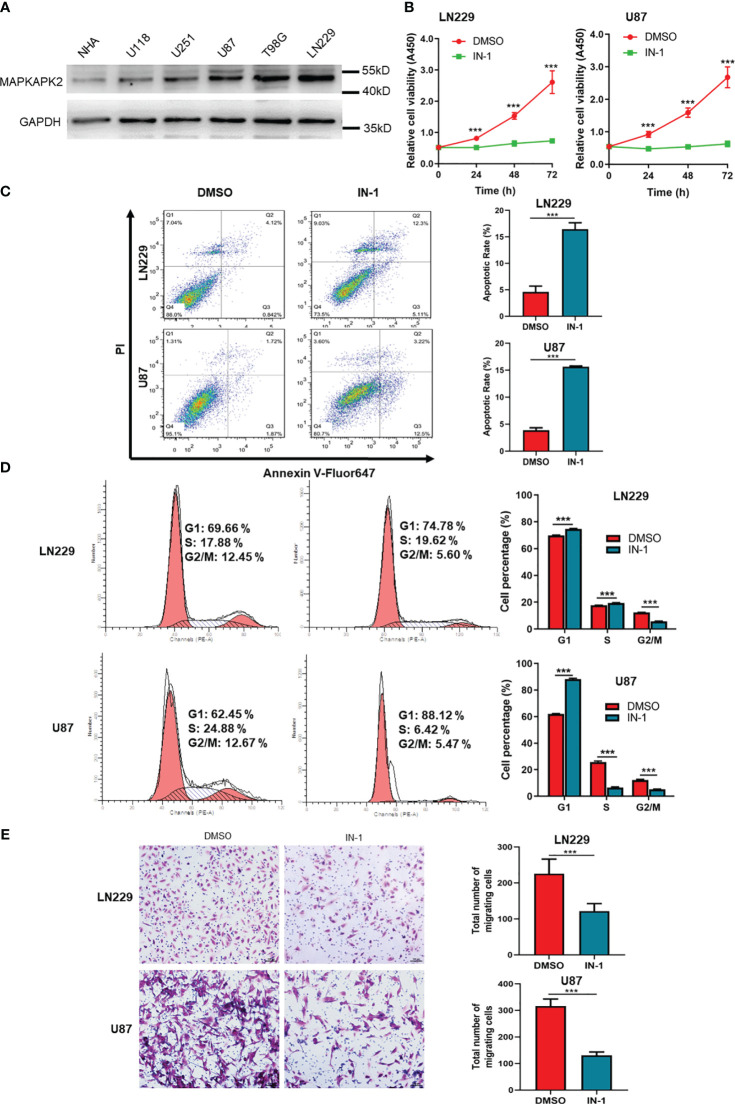
MAPKAPK2 promotes the proliferation and migration of GBM cells. **(A)** MAPKAPK2 protein expression in GBM cell lines U118, U251, U87, T98G, LN229, and the normal human astrocyte NHA detected by immunoblot. **(B)** Cell viability of U87 and LN229 cells with MAPKAPK2 inhibitor IN-1 detected by CCK8 (n = 15). **(C)** Representative images and statistical graphs of cell death rates in U87 and LN229 cells with MAPKAPK2 inhibitor IN-1 detected by FCM (n = 3). **(D)** The cell-cycle analysis by FCM in U87 and LN229 cells with MAPKAPK2 inhibitor IN-1 (n = 3). **(E)** The cell migration detected by Transwell chamber in U87 and LN229 cells (n = 5). Data are represented as the mean ± SD. Two-way ANOVA in **(B, D)** Student’s-*t* test in **(C)** ***p < 0.001.

In order to verify the promoting role of MAPKAPK2 in GBM cells, we performed the cell function detection including cell viability, cell death, cell cycle, and cell migration in GBM cells by inhibition of MAPKAPK2 with a selective inhibitor IN-1. We identified MAPKAPK2 protein levels in GBM cell lines U118, U251, U87, T98G, and LN229 by immunoblot (**Figure 7A**). MAPKAPK2 was prevalent in U87, T98G, and LN229 cells. U87 and LN229 were chosen to be used during the following experiments. The cell viability was significantly reduced, and the cell death rate obviously elevated with the MAPKAPK2 inhibitor in U87 and LN229 cells (**Figures 7B, C**). Cell cycle in U87 and LN229 cells was arrested in G1 with the MAPKAPK2 inhibitor detected by FCM (**Figure 7D**). Cell migration ability was also significantly reduced with MAPKAPK2 inhibition in U87 and LN229 cells (**Figure 7E**).

These data elucidate that MAPKAPK2 promotes the GBM progression by aggravating the proliferation and migration of GBM cells.

### 3.5 *MAPKAPK2* is mainly prevalent in tumor cells and macrophages in glioma by single-cell sequencing analyses

**Figure 8:**
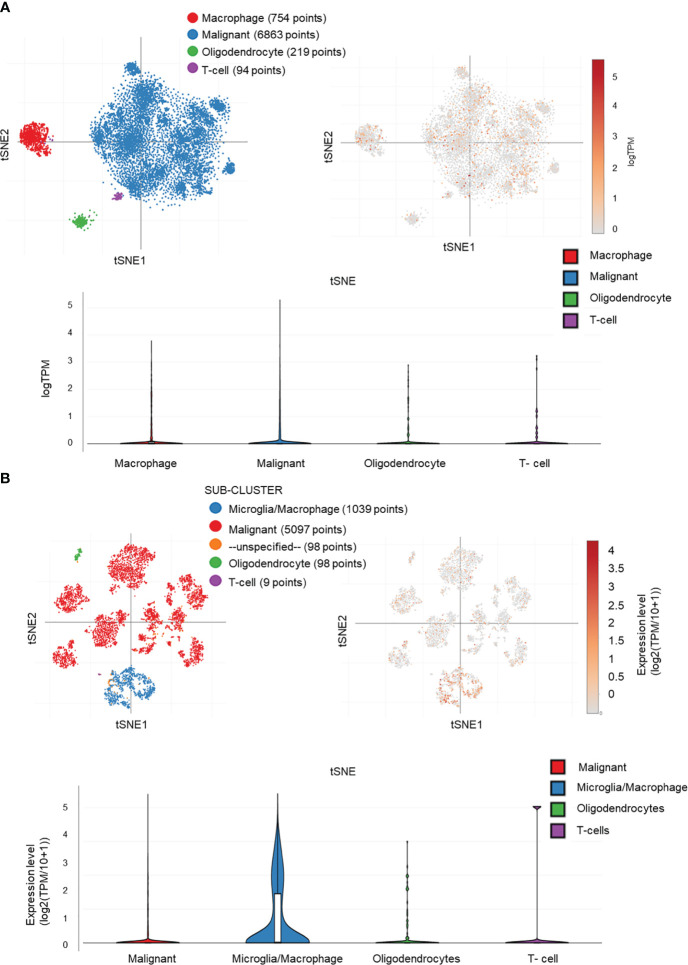
*MAPKAPK2* is mainly expressed in macrophages and tumor cells in glioma. **(A, B)** Cell types of *MAPKAPK2* expression analyses in single-cell RNA sequencing data from two studies (**A**, study 1, **B**, study 2).

We performed the single-cell sequencing analyses from TCGA to clarify the specific cell type of *MAPKAPK2* expression due to the heterogeneity and the complex components of glioma. Four diverse cell types, namely, microglia/macrophages, malignant tumor cells, oligodendrocytes, and T cells, in glioma were analyzed by single-cell sequencing analyses. *MAPKAPK2* were present in all of these four different types of cells, whereas the microglia/macrophages and malignant tumor cells were the mainly enriched cells, especially microglia/macrophages from two different datasets (**Figures 8A, B**), which was consistent with the finding in **Figure 2A**.

These data suggest that MAPKAPK2 may not only function in glioma cells but also probably be involved in the immune regulation in glioma tissue.

### 3.6 MAPKAPK2 is correlated with immune cell infiltration in glioma

**Figure 9:**
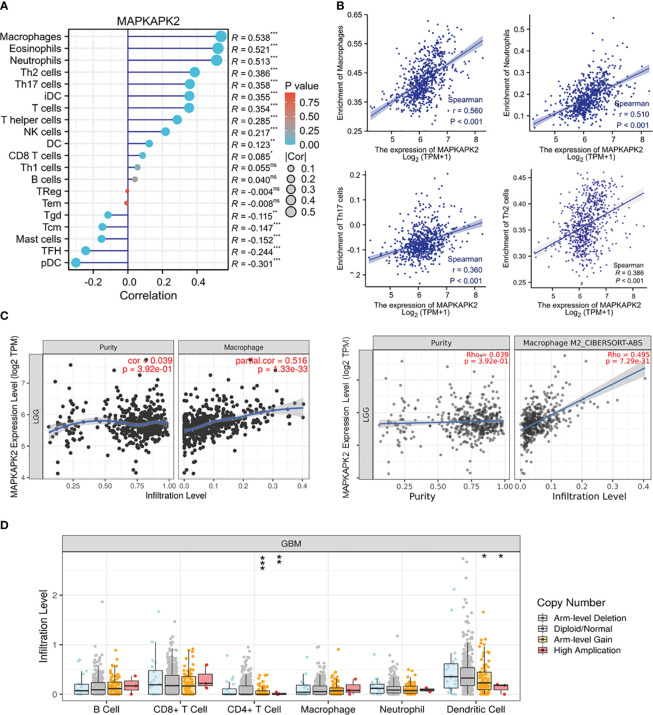
*MAPKAPK2* is correlated with immune cell infiltration in glioma. **(A)** Lollipop plots of the correlation analysis between the *MAPKAPK2* level and different immune cells in glioma from TCGA. The size of dots represents the absolute value of Spearman’s R. **(B)** Correlation analyses of *MAPKAPK2* with four different types of enriched immune cells. **(C)** Correlation analyses of *MAPKAPK2* with macrophage and M2 macrophage infiltration in LGG from TCGA. **(D)** Different infiltration levels of immune cells with different *MAPKAPK2* gene alterations in GBM analyzed by SCNA module. SCNAs were defined using GISTIC 2.0. *p<0.05, **p<0.01, ***p<0.001, ns, no significance.

In order to investigate the role of MAPKAPK2 in immune regulation, we firstly analyzed the association of MAPKAPK2 with diverse immune cells. We found that MAPKAPK2 was positively correlated with neutrophils, eosinophils, helper T (Th) 2 cells, and Th17 cells in total glioma (**Figures 9A, B**). In LGG, MAPKAPK2 expression was positively correlated with infiltration of macrophages, especially M2 macrophages (**Figure 9C**). A somatic copy number alteration (SCNA) module was constructed to analyze the immune infiltration distribution by the SCNA status of MAPKAPK2 in GBM. The infiltration levels of cluster of differentiation (CD) 4^+^ T cells and dendritic cells were significantly decreased in the MAPKAPK2 arm-level gain and high-amplification group compared with the normal group (**Figure 9D**).

These analyses indicated that MAPKAPK2 correlated with immune cell infiltration and may participate in the immune regulation of the glioma microenvironment.

### 3.7 MAPKAPK2 is correlated with immunomodulators, chemokines, and chemokine receptors in GBM

**Figure 10:**
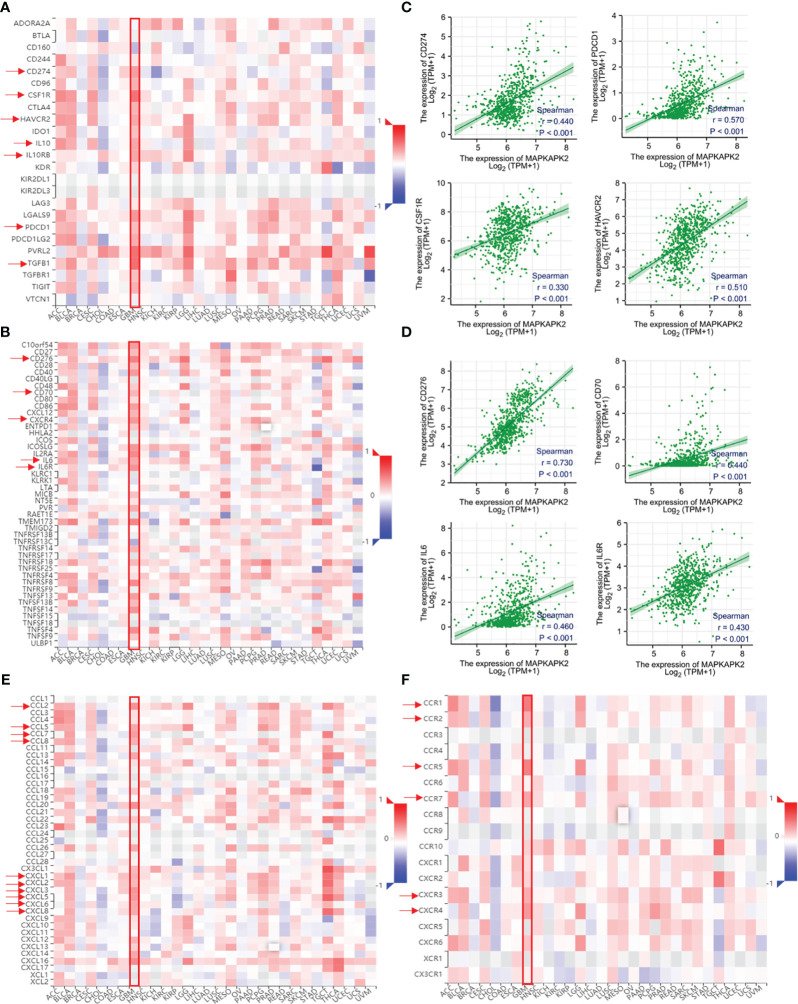
*MAPKAPK2* is correlated with important immune regulators, chemokines, and chemokine receptors in glioma tissues. **(A, B)** The heatmap of correlation analyses of MAPKAPK2 with important immune regulators and cytokines across 30 types of human tumors. **(C, D)** Correlation analyses of *MAPKAPK2* with immune regulating genes *CD274*, *PDCD1*, *CSF1R*, *HAVCR2* **(C)** and *CD276*, *CD70*, *IL6*, *IL6R* **(D)**. **(E, F)** The heatmap of correlation analyses of *MAPKAPK2* with chemokines **(E)** and chemokines receptors **(F)** across 30 types of human tumors.

In view of the function of MAPKAPK2 in glioma progression and the relationship with immune cell infiltration, the role of MAPKAPK2 in the regulation of the immune microenvironment in glioma is still obscure and the mechanism of promoting glioma progression remains unknown. We further analyzed the relationship of MAPKAPK2 with immunomodulator and chemokine and chemokine receptors. As shown in **Figure 10**, MAPKAPK2 was positively correlated with many immunoinhibitors such as CD274, programmed cell death protein (PDCD)1, transforming growth factor (TGF)-β1, IL10, IL10Rβ, colony-stimulating factor (CSF) 1R and hepatitis A virus cellular receptor (HAVCR)2; immunostimulators such as CD70, CD276, IL6, and IL6R (**Figures 10A–D**); chemokines such as chemokine C–C motif ligand (CCL) 2, CCL5, CCL7, CCL8, and chemokine C–X–C-motif ligand (CXCL) 1, 2, 3, 5, 6, 8; and chemokine receptors such as chemokine C–C-motif receptor (CCR)1, CCR2, CCR5, CCR7, chemokine C–X–C-motif receptor (CXCR) 3, and CXCR4 in GBM (**Figures 10E, F**).

Those results provided us the clues that MAPKAPK2 may function as a glioma promoting factor by the regulation of immune regulation molecules.

## 4 Discussion

The centerpiece treatment of glioma including surgery followed by radiation and adjuvant chemotherapy did not obviously improve the prognosis of glioma patients. Molecular target therapy and immune therapy are expected to improve the clinical outcomes of glioma patients (19, 20). Here, our findings suggested that MAPKAPK2 mRNA and protein levels are both significantly elevated in glioma especially GBM and MAPKAPK2 is positively correlated with poor prognosis of glioma patients. Cell cycle, cell adhesion, cytoskeleton regulation, DNA damage repair, and multiple immune regulation function are enriched in cell function and signaling pathways enrichment analyses. Meanwhile, MAPKAPK2 promotes GBM cell proliferation and migration in vitro. MAPKAPK2 is correlated with immune cell infiltration in glioma tissue. Mechanistically, MAPKAPK2 may regulate inflammatory cytokine, chemokine and chemokine receptor, and immune regulation molecule expression to facilitate glioma progression. The present data provided evidence and clues to a better understanding of the glioma promoting role of MAPKAPK2 and the underlying mechanisms in glioma progression. Targeting the MAPKAPK2 may supply a promising strategy for not only molecular-targeted therapy but also immunotherapy.

The survival analyses of subgroups of glioma indicated that high levels of MAPKAPK2 predict poor prognosis in grade 2 and grade 3 but not grade 4, which might be mainly caused by the limited number of patients with short survival time and low survival rate enrolled in the grade 4 cohort. Molecular markers with great significance in prognosis evaluation of glioma were referred into the classification of molecular subtypes of glioma in the tumor classification of central nervous system WHO 5^th^ (21). MAPKAPK2 is prevalent in IDH wt and 1p/19q non-codeletion glioma patients and is correlated with poor prognosis of glioma patients. More than 95% primary GBM and around 20%–30% low-grade gliomas are IDH wt (22). Correspondingly, there was also no significance in the survival analysis in IDH wt patients upon MAPKAPK2 expression. 1p/19q codeletion usually occurs in low-grade glioma and confers favorable prognosis of glioma patients among diffuse glioma (23). MAPKAPK2 predicts poor prognosis of glioma patients in the 1p/19q non-codeletion cohort but not in the codeletion cohort, which suggests that MAPAPK2 may assist in the prognosis evaluation of the glioma patients with 1p/19q non-codeletion. Therefore, MAPKAPK2 is a valuable marker for the prognosis evaluation of glioma including the IDH mutant and 1p/19q non-codeletion cohorts.

The biological function of MAPKAPK2 in tumor has not been well elucidated. MAPKAPK2 promotes the proliferation of head and neck squamous cell carcinoma (6), the metastasis of breast cancer (24), and the invasion of bladder cancer (8). Here, cell cycle, cell adhesion, and regulation of cytoskeleton are enriched upon MAPKAPK2 expression. Consistently, we demonstrated that MAPKAPK2 promotes the GBM cell proliferation and migration, which demonstrated that GBM cell intrinsic MAPKAPK2 enhances the malignant behavior of GBM cells.

Previous studies reported that MAPKAPK2 was involved in the chemotherapy or radiotherapy resistance in pancreatic cancer cells (11, 25) and breast cancer cells (13). MAPKAPK2 is significantly correlated with DNA repair, DNA double-strand break repair, base excision repair, and DNA damage checkpoint in GSEAs. Temozolomide (TMZ) is the first-choice chemotherapeutic in the treatment of GBM and astrocytoma. However, intrinsic and acquired TMZ resistance usually leads to the failure of GBM therapy (26). TMZ exerts its antitumor effect by inducing the DNA damage and the tumor cell-programmed cell death. DNA damage repair pathways can obviously affect the reactivity of tumor cells to chemotherapeutics. Here, we speculated that MAPKAPK2 may be involved in the chemotherapy or radiotherapy resistance in glioma and more evidence may be needed to demonstrate the potential role of MAPKAPK2.

Bioinformatic enrichment analyses revealed the related function of MAPKAPK2 in immune regulation. MAPKAPK2 is the direct substrate of p38. The p38–MAPKAPK2 signaling axis potentiated the expression of some transcription factors of oncogenes and the downstream proteins to secrete inflammation cytokines and chemokines after activated by cellular or environmental stressors (27, 28). Activated MAPKAPK2 is found in more than 50% of investigated GBM tissues (17). As reported, MAPKAPK2 promotes colon tumor growth by regulating macrophage chemokine activity and recruitment (14). Our data showed that MAPKAPK2 is mainly prevalent in macrophages and glioma cells and is positively correlated with the macrophage infiltration in glioma. Additionally, MAPKAPK2 is positively correlated with a series of chemokines or chemokine receptors, which might be the driving force of the recruit of macrophages or other immune cells. However, MAPKAPK2 is positively correlated with a series of immune regulators such as CD274, CD276, PDCD1, and CD70, which probably affect the tumor killing effect of the infiltrated immune cells in glioma tissue. In addition to these, MAPKAPK2 is positively correlated with IL6, IL6R, IL10, IL10RB, TGFβ1, etc., in the present study, which may facilitate the glioma progression. IL6 promoted the proliferation of GBM cells, and a high level of IL6 predicted poor prognosis of glioma (29). As reported, IL10 secreted by glioma cells are thought to activate tumor-infiltrating immune cells such as microglia and macrophages, which would produce more IL-10 to compose the majority in glioma tissue. IL10 is reported to support immune evasion in CD133^+^ stem-like glioma cells (30). The combination of IL10 with its receptor IL10R expressed in glioma cells would further promote the proliferation, migration, and invasion of glioma. Enriched MAPKAPK2 expression in microglia/macrophages and glioma cells might facilitate the production of IL10 to promote glioma progression (31). The role of MAPKAPK2 in inflammation and immune regulation in glioma tissue may supply a new angle of view to glioma therapy.

In summary, aberrantly expressed MAPKAPK2 is positively correlated with poor prognosis of glioma. MAPKAPK2 was found to aggravate the proliferation and migration of glioma, and it was closely correlated with DNA damage repair and immune regulation in glioma tissue. Our findings provided evidence that MAPKAPK2 may be a valuable target for evaluating glioma prognosis and developing clinical therapy.

## Data availability statement

The original contributions presented in the study are included in the article/supplementary material. Further inquiries can be directed to the corresponding author.

## Ethics statement

The studies involving humans were approved by the Ethics Committee of the Affiliated Hospital of Xuzhou Medical University. The studies were conducted in accordance with the local legislation and institutional requirements. The participants provided their written informed consent to participate in this study.

## Author contributions

JS: Conceptualization, Funding acquisition, Resources, Writing – original draft, Data curation, Formal Analysis, Investigation, Methodology, Validation. SW: Formal Analysis, Methodology, Writing – original draft, Investigation, Resources, Validation. WZ: Formal Analysis, Methodology, Writing – review & editing, Investigation, Resources. SX: Formal Analysis, Methodology, Writing – review & editing, Investigation, Resources. LZ: Resources, Writing – review & editing, Project administration, Validation. JR: Conceptualization, Funding acquisition, Project administration, Supervision, Writing – original draft, Writing – review & editing, Resources, Validation.
